# Palearctic elements in the old world tropics: a taxonomic revision of the ant genus *Temnothorax* Mayr (Hymenoptera, Formicidae) for the Afrotropical biogeographical region

**DOI:** 10.3897/zookeys.483.9111

**Published:** 2015-02-20

**Authors:** Matthew Prebus

**Affiliations:** 1 Department of Entomology & Nematology, University of California Davis, Davis, CA 95616

**Keywords:** Afrotropical region, Formicoxenini, Kenya, Laikipia, Sudan, taxonomy, *Temnothorax*

## Abstract

Four new Afrotropical species of the ant genus *Temnothorax* are described and illustrated, all from Kenya. Based upon high resemblance to taxa known from the North African and Iberian territories of the Mediterranean region, these new tropical elements are placed into known Palaearctic species complexes. Specifically, *Temnothorax brevidentis* **sp. n.**, *Temnothorax mpala* **sp. n.** and *Temnothorax rufus* **sp. n.** are placed in the *laurae* species group, and *Temnothorax solidinodus* **sp. n.** is placed in the *angustulus* species group. Two already known *Temnothorax* species from the region, *Temnothorax cenatus* (Bolton, 1982) and *Temnothorax megalops* (Hamann & Klemm, 1967), are also placed into the *laurae* species group based on the high number of shared morphological characters. Diagnoses for the African representatives of *laurae* and *angustulus* species groups of the Afrotropical biogeographical region are provided. A key to workers of the six *Temnothorax* species known to occur in the Afrotropical biogeographical region is provided, as well as diagnoses of morphologically similar myrmicine genera.

## Introduction

*Temnothorax* Mayr, 1861 is a genus of small, generally inconspicuous ants found in habitats ranging from deserts to tropical rainforests. Many of the species belonging to this genus are positively thigmotactic, nesting in preformed cavities such as the shells of rotten nuts, beetle-carved chambers in wood, between cracks in stone, and in the soil. Colonies are typically quite small, often with fewer than 100 workers per nest (Beckers et al. 1989). The diet and foraging habits of these ants are mostly unknown, but they are suspected to be trophic generalists; a few studies have recorded instances of granivory and consumption of elaiosomes (Espadaler 1997, Fokuhl et al. 2012).

With more than 350 named species (Bolton 2014), *Temnothorax* is a large genus with a predominantly Holarctic distribution. A few notable exceptions to this general distribution include a large radiation in Meso-America, including the islands of the Caribbean (Kempf 1959; Baroni-Urbani 1978), two undescribed species from mountainous northern Vietnam (Eguchi 2011) and two described species from sub-Saharan Africa (Bolton 1982). Below, four new species of *Temnothorax* are described from the latter region, all from within Kenya.

In the last revision of the genus *Leptothorax* of the Afrotropical biogeographic region, Bolton (1982) described three new species, bringing the number of described species to 11. In 2003 Bolton revived the genera *Temnothorax* and *Nesomyrmex* from synonymy with *Leptothorax*; nine of the Afrotropical species were transferred to *Nesomyrmex*, while the remaining two were transferred to *Temnothorax*. One of these species, *Temnothorax megalops*, has been noted by several authors to have close affinities to members of the *laurae* species group (Emery 1884, Tinaut 1995, Cagniant and Espadaler 1997), predominantly of northern Africa and southern Europe. Although a comprehensive revision of this group is beyond the scope of this paper, I propose that *Temnothorax mpala* sp. n. is also a member of this group, differing from most of the other members by the absence of a distinct metanotal groove. The species of *Temnothorax cenatus*, *Temnothorax brevidentis* and *Temnothorax rufus* also appear to be members of this group, especially when considering *Temnothorax crepuscularis* and *Temnothorax caesari* of the Iberian peninsula.

The paucity of *Temnothorax* species in the Afrotropics led Bolton (1982) to speculate that this may be a result of direct competition from members of the genus *Tetramorium*, which are extremely diverse in sub-Saharan Africa. The genera *Nesomyrmex* and *Tetramorium*, which are not particularly closely related to *Temnothorax* (Ward et al. 2014), are often confused with this genus due to convergence in general habitus. Table 1, updated from Bolton 1982, will aid in separating these genera from *Temnothorax*.

**Table 1.** Diagnostic characters of three sympatric and morphologically similar myrmicine genera.

Character	*Temnothorax*	*Nesomyrmex*	*Tetramorium*
Sting with apical or apicodorsal lamelliform appendage	no	no	yes
Maxillary palp segments	5	5	3 or 4
Lateral clypeal lobes raised into a narrow ridge or shield-wall in front of antennal insertions	no	no	yes
Median clypeal lobe in the form of an apron that fits tightly over the base of the mandibular dorsum in profile	no	yes	no
Number of mandibular teeth	5	5	6 to 7

### Abbreviations of depositories

The collection abbreviations follow Evenhuis (2009). The material upon which this study is based is located and/or was examined at the following institutions:

BMNH The Natural History Museum (British Museum, Natural History), London

CASC California Academy of Sciences, San Francisco, USA

HLMD Hessiches Landemuseum, Darmstadt, Germany

LACM Los Angeles County Museum of Natural History, Los Angeles, USA

MHNG Muséum d’Histoire Naturelle de la Ville de Genève, Geneva, Switzerland

NHMW Naturhistorisches Museum Wien, Vienna, Austria

## Material and methods

The material used in this study is relatively scarce. Much of it has come from collections made by Roy Snelling at the LACM. The type material of the new species and all imaged specimens can be uniquely identified with specimen-level codes affixed to each pin (e.g. CASENT0078328). Types of all new species described below will be deposited at the institutions mentioned above. Digital color images were created using a JVC KY-F75 digital camera and Syncroscopy Auto-Montage software (version 5.0), or a Leica DFC 425 camera in combination with the Leica Application Suite software (version 3.8). All images presented are available online and can be seen on AntWeb (http://www.antweb.org).

**Figure 1.**
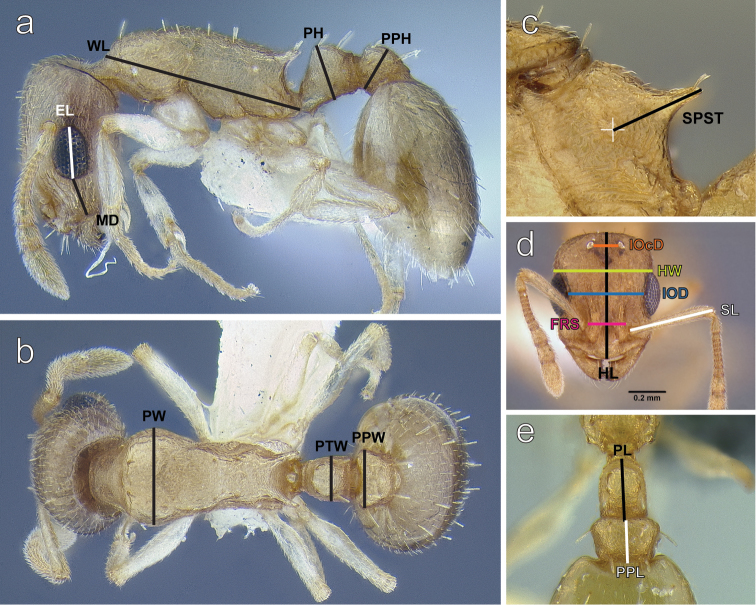
**a** *Temnothorax mpala* sp. n. worker (LACMENT323183) in lateral view illustrating measurements used: malar distance (MD), eye length (EL) Weber’s length (WL), petiole height (PH), postpetiole height (PPH) **b** *Temnothorax mpala* sp. n. worker (LACMENT323183) in dorsal view: pronotal width (PW), petiole width (PTW), postpetiole width (PPW) **c** *Temnothorax mpala* sp. n. dealate gyne (LACMENT323184) in lateral view illustrating propodeal spine length (SPST) **d** *Temnothorax mpala* sp. n. dealate gyne (LACMENT323183) in full face view: interocellar distance (IOcD), head width (HW), interocular distance (IOD), frontal carina distance (FRS), scape length (SL) **e** *Temnothorax mpala* sp. n. worker (LACMENT323183) in dorsal view: petiole length (PL), postpetiole length (PPL).

The measurements and indices used in this study are based on Bolton (1982), Radchenko (2004), Hita Garcia and Fisher (2011), Schulz et al. (2007), Seifert (2006), and Bharti et al. (2011). The measurements were taken with a Leica MZ 12.5 equipped with an orthogonal pair of micrometers at a magnification of up to 115×. Measurements and indices are presented as minimum and maximum values with arithmetic means in parentheses. In addition, all measurements are expressed in mm to three decimal places. See Figure 1 for illustrations of the following measurements.

EL Eye length: maximum diameter of compound eye including all structurally visible ommatidia irrespective of the pigmentation status, measured in oblique lateral view.

FRS Distance of the frontal carinae immediately caudal of the posterior intersection points between frontal carinae and the lamellae dorsal of the torulus. If these dorsal lamellae do not laterally surpass the frontal carinae, the deepest point of scape corner pits may be taken as reference line. These pits take up the inner corner of scape base when the scape is fully switched caudad and produce a dark triangular shadow in the lateral frontal lobes immediately posterior of the dorsal lamellae of scape joint capsule (Seifert 2006).

HL Head length: maximum distance from the mid-point of the anterior clypeal margin to the mid-point of the posterior margin of head, measured in full-face view. Impressions on anterior clypeal margin and posterior head margin reduce head length.

HW Head width: width of head directly behind the eyes, measured in full-face view.

IOcD Inter-ocellar distance: minimum distance between the posterior-most pair of ocelli. Applies to queens and males.

IOD Inter-ocular distance: minimum distance between the compound eyes, measured in full-face view.

MD Malar distance: the minimum distance between the anterior margin of the compound eye and the base of the mandible

PH Petiole height: The maximum height of the petiole, measured from the apex of the node to ventral edge of petiole, parallel to the anterior margin of the petiole (Bharti et al. 2011).

PL Petiole length: the maximum length of the petiole is measured in dorsal view from the anterior notch close to the propodeum to the articulation with the postpetiole. Both points must be in focus (Schulz et al. 2007).

PPH Postpetiole height: maximum height of the postpetiole measured in lateral view from the highest (median) point of the node to the ventral outline. The measuring line is placed at an orthogonal angle to the ventral outline of the node.

PPL Postpetiole length: maximum length of postpetiole measured in dorsal view, excluding helcium.

PPW Postpetiole width: maximum width of postpetiole measured in dorsal view.

PTW Petiole width: maximum width of petiole measured in dorsal view.

PW Pronotal width: maximum width of pronotum measured in dorsal view.

SL Scape length: maximum scape length excluding basal condyle and neck.

SPST Distance between the center of the propodeal stigma and spine tip. The stigma center refers to the midpoint defined by the outer cuticular ring but not to the center of stigma opening, which may be positioned eccentrically (Seifert 2006).

WL Weber’s length: diagonal length of mesosoma in lateral view from the postero-ventral margin of propodeal lobe to the anterior-most point of pronotal slope, excluding the neck.

Indices

CI Cephalic index: HW / HL × 100

DPeI Dorsal petiole index: PTW / PTL × 100

DPpI Dorsal postpetiole index: PPW / PPL × 100

LPpI Lateral postpetiole index: PPL / PPH × 100

OI Ocular index: EL / HW × 100

PeNI Petiolar node index: PTW / PW × 100

PPI Postpetiole index: PPW / PTW × 100

PpNI Postpetiolar node index: PPW / PW × 100

PSLI Propodeal spine index: SPST / HL × 100

SI Scape index: SL / HL × 100

The varying degree of inclination of pubescence and pilosity are often of high diagnostic value throughout a broad spectrum of ant genera. In this context I use the terms “erect”, “suberect”, “subdecumbent”, “decumbent”, and “appressed” following Wilson (1955).

### Species concept

Due to the severe paucity of specimens of this genus from tropical Africa, especially of entire nest collections, the species concept used in this article is loosely based on the biological species concept. The specimens described in this article, based upon their close geographical proximity and their distinct morphology in relation to each other, hypothetically represent reproductively isolated species.

### Species-group affinities

Many of the *Temnothorax* species of sub-Saharan Africa bear close morphological resemblance to the ants of the *laurae* species group of North Africa and southern Europe as described by Cagniant and Espadaler 1997, primarily in having large eyes and, with the exception of *Temnothorax mpala* sp n., bearing a metanotal groove. *Temnothorax cenatus*, *Temnothorax brevidentis* sp. n. and *Temnothorax rufus* sp. n. are morphologically similar to members of the *laurae* group from the Iberian peninsula in their coarsely striate sculpture and impressed metanotal groove. *Temnothorax mpala* sp. n. and *Temnothorax megalops* appear to be closely related to one another, differing mainly in the morphology of the head and mesosoma. However, none of the *laurae*-group species presented in this article display micropilosity between the facets of the compound eye, which is one of the distinguishing features of this species group on the Iberian peninsula and North Africa. *Temnothorax solidinodus* sp. n., on the other hand, bears a close resemblance to *Temnothorax angustulus* of the North African and Iberian regions. Differing primarily in the morphology of the petiolar node (dorsally angulate in *Temnothorax angustulus*, massive and blunt dorsally in *Temnothorax solidinodus* sp. n.), both species are apparently arboreal.

### Diagnosis of the species groups of *Temnothorax* in the Afrotropical region

*laurae* group: Eyes large relative to the length of the head capsule: OI > 30. Head capsule elongate: CI < 85. Postpetiole more or less trapezoidal in dorsal view; widest anterior to the midlength of the segment. Antennal scape variable relative to head capsule length: 68 < SI > 97.

*angustulus* group: Eyes small relative to the length of the head capsule: OI < 30. Head capsule nearly equal in maximum length and width: CI > 85. Postpetiole widest at the midlength of the segment in dorsal view. Antennal scape short relative to head capsule length: SI > 70.

### Biogeographical notes

Due to close affinities to the species of the Mediterranean and southern Palearctic biogeographical region, the species presented in this article are most likely relict fauna which may have become isolated in the sub-Saharan region following the African Humid Period (deMenocal et al. 2000) or a similar event, during which the Sahara desert was mostly vegetated. Alternatively, the ancestral species of the present fauna may have migrated from Northern Africa into sub-Saharan region via the Great Rift Valley. Subsequently, these species may have been prevented from radiating in the African tropics due to the ecologically similar, diverse and successful species of the genus *Tetramorium*, as hypothesized by Bolton (1982).

## Synopsis of Afrotropical *Temnothorax* species

*Temnothorax brevidentis* Prebus, **sp. n.**

*Temnothorax cenatus* (Bolton, 1982)

*Temnothorax megalops* (Hamann & Klemm, 1967)

*Temnothorax mpala* Prebus, **sp. n.**

*Temnothorax rufus* Prebus, **sp. n.**

*Temnothorax solidinodus* Prebus, **sp. n.**

### Key to the afrotropical *Temnothorax* based on workers

**Table:** 

1	Compound eyes moderate in size: OI < 30. Head square CI > 8	***Temnothorax solidinodus* sp. n.**
–	Compound eyes large: OI > 30. Head elongate CI < 85	**2**
2	In full-face view antennal scapes short, distinctly failing to reach posterior margin of head (Fig. 2a); SI < 80. In dorsal view postpetiole trapezoidal, widest at the anterior 1/4 of the segment (Fig. 2c)	**3**
–	In full face view antennal scapes long, surpassing posterior margin of head by at least the length of the first funicular segment (Fig. 2b); SI > 80. In dorsal view, postpetiole widest at the anterior 1/3 of the segment (Fig. 2d)	**4**
3	Metanotal groove present (Fig. 3a); posterior margin of head shallowly but distinctly impressed (Fig. 3c)	***Temnothorax megalops* (Hamman & Klemm)**
–	Metanotal groove absent (Fig. 3b), barely visible as an indistinct break in the sculpture dorsally in some specimens; posterior margin of head flat (Fig. 3d)	***Temnothorax mpala* sp. n.**
4	Propodeal spines short: PSLI < 23 (Fig. 4a)	***Temnothorax brevidentis* sp. n.**
–	Propodeal spines moderately long and acute: PSLI > 23 (Fig. 4b)	**4**
5	Antennal scapes long; surpassing the posterior margin of the head by the length of the first two funicular segments when fully retracted: SI 94–97 (Fig. 4c)	***Temnothorax rufus* sp. n.**
–	Antennal scapes shorter; surpassing the posterior margin of the head by the length of the first funicular segment when fully retracted: SI 84 (Fig. 4d)	***Temnothorax cenatus*** (**Bolton)**

**Figure 2.**
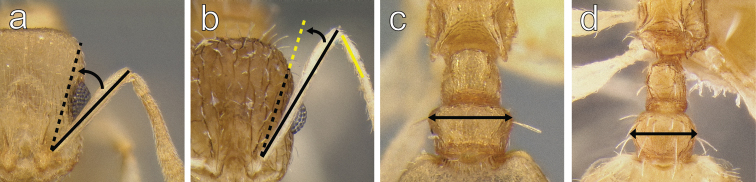
**a** *Temnothorax mpala* sp. n. (LACMENT323183) in full face view **b** *Temnothorax rufus* sp. n. (CASENT0712675) in full face view **c** *Temnothorax mpala* sp. n. (LACMENT323183) in dorsal view **d** *Temnothorax rufus* sp. n. (CASENT0712675) in dorsal view.

**Figure 3.**
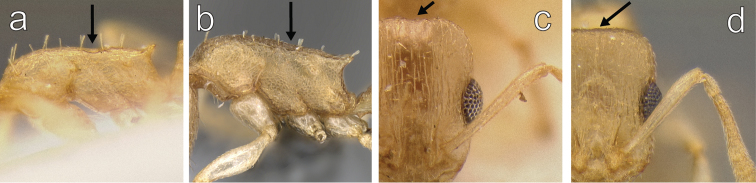
**a** *Temnothorax megalops* (CASENT0712601) in lateral view **b** *Temnothorax mpala* sp. n. (LACMENT323183) in lateral view **c** *Temnothorax megalops* (CASENT0712601) in full face view **d** *Temnothorax mpala* (LACMENT323183) in full face view.

**Figure 4.**
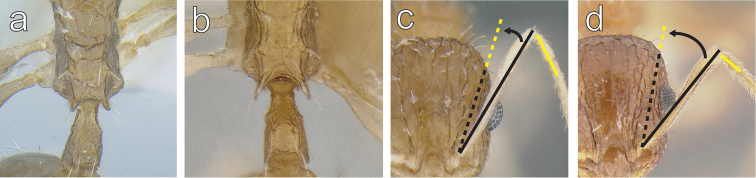
**a** *Temnothorax brevidentis* sp. n. (CASENT0712603) in dorsal view **b** *Temnothorax cenatus* (CASENT0900308) in dorsal view **c** *Temnothorax rufus* sp. n. (CASENT0712675) in full face view **d** *Temnothorax cenatus* (CASENT0900308) in full face view.

#### Temnothorax brevidentis

Taxon classificationAnimaliaHymenopteraFormicidae

Prebus sp. n.

http://zoobank.org/F4C3293E-92D1-4AA9-9473-BFC285D2E0D8

Figs 4a , 5

##### Type material.

Holotype worker, KENYA, Laikipia District, Mpala Research Centre, 1650 m, 0.29°N, 36.90°E, Acacia woodland, stray in litter, collection code #99-056, 24.IX.1999 (*R.R. Snelling*) (BMNH: CASENT0712603).

##### Diagnosis.

*Temnothorax brevidentis* is easily distinguishable from the other Afrotropical species by the following character combination:

Antennal scapes surpassing the posterior margin of the head by the length of the first funicular segment; postpetiole widest at the anterior 1/3 of the segment; setae on the posterior margin of the first gastric tergite separated by about their own length; posterior margin of head rounded; metanotal groove shallowly impressed; compound eyes moderate in size; propodeal spines short.

##### Worker measurements

**(n = 1).** EL 0.155; FRS 0.185; HL 0.633; HW 0.479; IOD 0.422; IOcD N/A; MD 0.172; PH 0.186; PL 0.243; PPH 0.196; PPL 0.196; PPW 0.245; PTW 0.144; PW 0.355; SL 0.573; SPST 0.136; WL 0.802.

**Indices:** CI 75.7; DPeI 59.3; DPpI 125; LPeI 131; LPpI 100; OI 32.4; PeNI 40.6; PpNI 69; PPI 170; PSLI 21.5; SI 90.5.

##### Worker description.

Head longer than wide (CI 76); head sides parallel, but converging toward the mandibular insertions anteriorly beyond the level of the antennal insertions in full-face view; posterior head margin convex and posterior corners of head broadly rounded. Anterior clypeal margin convex and entire, with the median clypeal lobe projecting slightly beyond the lateral clypeal lobes. Frontal carinae developed: extending posteriorly about one-half the length of the compound eye. Antennae 12-segmented,antennal scapes relatively long, extending past the posterior margin of the head by the length of the first funicular segment (SI 120). Eyes moderate in size (OI 32), 8 ommatidia in longest row.

Mesosoma relatively slender (WL 1.26 times HL); promesonotal suture absent. Metanotal groove narrowly and shallowly impressed. Propodeal spines short and acute (PSLI 21.5); propodeal lobes small and rounded.

Petiole without a differentiated peduncle. In profile, the anterior face of node forming a shallow concavity anteriorly as it joins the anterior portion of the petiole. Petiolar node in profile relatively low (LPeI 131), and uniformly rounded, without distinct angles between anterior, dorsal, and posterior faces. In dorsal view petiole elongate (DPeI 59). Postpetiole in profile globular (LPpI 100) and roughly the same height as the petiole. Postpetiole in dorsal view transversely elongate-oval, widest at 1/3 of the total postpetiole length from anterior margin (DPpI 125), widest at 1/3 of the total postpetiole length from the anterior margin, and 1.7 times wider than petiole (PPI 170).

Mandibular sculpture: longitudinally striate along entire length. Clypeus smooth and shiny, bearing 9 longitudinal rugae, with median ruga strongly developed and running posteriorly from the anterior clypeal margin to the level of antennal insertions before weakening. Cephalic dorsum smooth and shining, with overlying well developed, widely spaced longitudinal rugae, which develop cross-linking rugae on the vertex. In profile, head coarsely reticulate; genae with irregular punctures anterior to the compound eye. Sculpture of mesosoma in dorsal view coarsely rugo-reticulate, with interspaces shining. Space between propodeal spines with a single well developed, arcuate transverse carina, which divides the propodeal dorsum from the declivity. Propodeal declivity irregularly shallowly punctate, bordered laterally by weak carinae which run from the ventral margins of the propodeal spines to the propodeal lobes. In profile, mesosoma predominantly finely rugo-reticulate; pronotum with coarse longitudinal rugae. Petiole and postpetiole finely punctate, with fine reticulation present on the dorsal surface of the petiolar node, and faint longitudinal rugae on the postpetiole. Gaster smooth and shining except for small, widely spaced piligerous punctures.

Head and mandibles nearly uniformly covered in a fine, yellowish pubescence. Dorsal surface of the head, including clypeus, frons and posterior margin of the head equipped with long, blunt-tipped setae. Anterior clypeal margin with two pairs of long setae flanking the median carina. Antennal scapes with short, sharp-tipped suberect pilosity. Pronotal “neck” and pronotal humeri with short, fine yellowish pubescence. Propleurae and procoxae with long, flexous, sharp-tipped pilosity. Dorsal surfaces of mesosoma, waist segments and gaster with uniformly erect, moderately long, abundant and blunt-tipped setae, their bases spaced from each other by the length of the setae or less. Bases of the setae on the posterior margin of first gastral tergite separated by about the length of the setae arising from them. Ventral surface of the gaster with sparse pilosity like that of the propleuron. Ventral surface of the post petiole free of pilosity.

**Figure 5.**
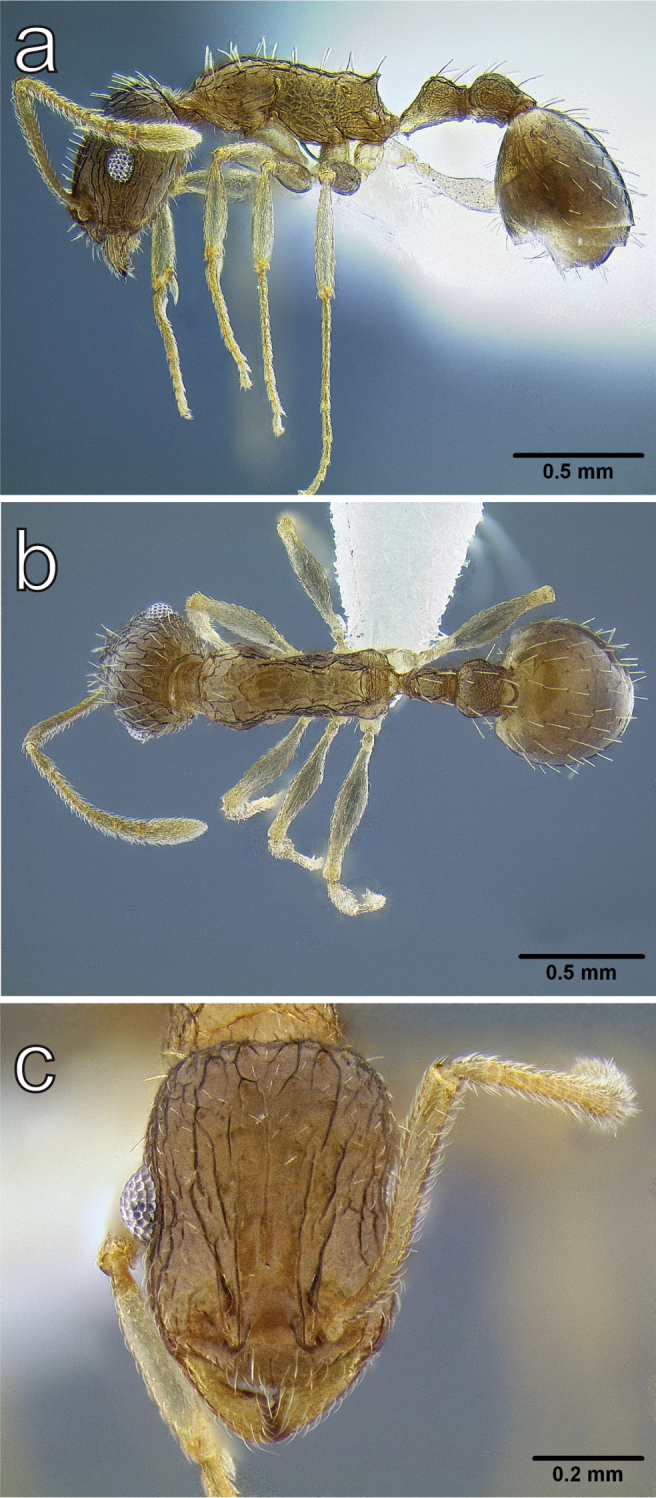
*Temnothorax brevidentis* sp. n., worker (CASENT0712603) **a** body in lateral view **b** body in dorsal view **c** head in full face view.

##### Queen.

Unknown

##### Male.

Unknown

##### Color.

Worker: Overall light reddish brown, with head and gaster slightly darker.

##### Distribution and ecology.

*Temnothorax brevidentis* is known only from acacia woodland leaf litter at the type locality, Mpala Research Centre.

##### Taxonomic notes.

*Temnothorax brevidentis* appears to be very closely related to both *Temnothorax cenatus* and *Temnothorax rufus*, differing primarily from these in having short propodeal spines.

#### Temnothorax cenatus

Taxon classificationAnimaliaHymenopteraFormicidae

(Bolton, 1982)

Figs 4b and d ; 6

Leptothorax cenatus : Bolton 1982: 327.
Temnothorax cenatus : Bolton 2003: 271.

##### Type material.

Holotype worker, KENYA, Lake Nakuru National Park, leaf litter, 6.XI.1974 (*V. Mahnert*) (MHNG) [examined]. Paratype, 1 worker from KENYA, Nakuru, Lake Elmenteita, 1800 m, 7.XI.1977 (*V. Mahnert & J.-L. Perret*) (BMNH: CASENT0900308) [examined].

##### Diagnosis.

The following character combination distinguishes *Temnothorax cenatus* from the other Afrotropical genus members:

Antennal scapes surpassing the posterior margin of the head by the length of the first funicular segment; postpetiole widest at the anterior 1/3 of the segment; posterior margin of head rounded; metanotal groove shallowly impressed; compound eyes moderate in size; propodeal spines moderately long.

##### Worker measurements

**(n = 1).** EL 0.174; FRS 0.222; HL 0.698; HW 0.558; IOD 0.468; IOcD N/A; MD 0.184; PH 0.219; PL 0.29; PPH 0.211; PPL 0.209; PPW 0.281; PTW 0.172; PW 0.398; SL 0.584; SPST 0.195; WL 0.922.

**Indices:** CI 79.9; DPeI 59.3; DPpI 134; LPeI 132; LPpI 99.1; OI 31.2; PeNI 43.2; PpNI 70.6; PPI 163; PSLI 27.9; SI 83.7.

##### Worker description.

Head longer than wide (CI 79.9); head sides parallel, but converging toward the mandibular insertions anteriorly beyond the level of the antennal insertions in full-face view; posterior head margin broadly convex and posterior corners of head broadly rounded. Anterior clypeal margin convex, with the median clypeal lobe projecting slightly beyond the lateral clypeal lobes. Frontal carinae developed: extending posteriorly to about midlength of the compound eye, after which they become indistinguishable from the ground rugulae of the head. Antennae 12-segmented,antennal scapes relatively long, surpassing the posterior margin of the head by about the length of the first funicular segment (SI 105). Eyes moderate in size (OI 31.2), with 11 ommatidia in longest row.

Mesosoma relatively slender (WL 1.32 times HL); promesonotal suture not impressed, barely visible as a darkened line in dorsal view, but not indicated by a break in the sculpture. Metanotal groove shallowly impressed; visible as a broad, shallow concavity in lateral view. Propodeal spines acute and moderately long (PSLI 27.9); propodeal lobes small and rounded.

Petiole without a differentiated peduncle. In profile, petiole with a low carina running transversely from the petiolar spiracle to the posterior margin; the anterior face of node forming a shallow concavity anteriorly as it joins the anterior portion of the petiole. Petiolar node in profile relatively low, with anterior and posterior faces broadly rounded (LPeI 132). In dorsal view petiole elongate (DPeI 59.3). Postpetiole in profile globular, nearly equal in height to petiolar node and relatively elongate (LPpI 99.1); in dorsal view transversely elongate-oval, widest at 1/3 of the total postpetiole length from anterior margin (DPpI 134) and 1.6 times wider than petiole (PPI 163).

Mandibular sculpture: distinctly longitudinally striate along entire length. Clypeus smooth and shiny, bearing 9 longitudinal rugae, with median ruga strongly developed and running posteriorly from the anterior clypeal margin to the level of antennal insertions before weakening. Cephalic dorsum predominantly longitudinally rugose, with transverse rugae incompletely connecting longitudinal rugae. In profile, sides of head coarsely rugo-reticulate; coarse punctures visible between rugae, particularly postero-ventrally to the compound eye. Sculpture of mesosoma in dorsal view with predominately longitudinal rugae on pronotum and mesonotum, becoming increasingly reticulate on the propodeum. Space between propodeal spines with several fine transverse rugae, propodeal declivity finely punctate. In profile, mesosoma rugo-reticulate; longitudinal rugae stronger on pronotum, becoming increasingly reticulate on mesopleuron, and giving way to coarse punctation on metapleuron. Petiole and postpetiole finely punctate, with weak overlying rugosity. Gaster smooth and shining except for small, widely spaced piligerous punctures.

Mandibles, lateral and ventral regions of the head nearly uniformly covered in a fine, yellowish pubescence. Dorsal surface of the head, including clypeus, frons and posterior margin of the head equipped with long, blunt-tipped setae. Anterior clypeal margin with two pairs of long setae flanking the median carina. Antennal scape pilosity abundant, sharp-tipped and decumbent.

Pronotal “neck” and pronotal humeri with short, fine yellowish pubescence. Propleurae and upper half of procoxae with long, flexous, sharp-tipped pilosity. Dorsal surfaces of mesosoma, waist segments and gaster with uniformly erect, moderately long, abundant and blunt-tipped setae, their bases spaced from each other by the length of the setae or less. Ventral surfaces of the post-petiole and gaster with sparse pilosity like that of the propleuron.

**Figure 6.**
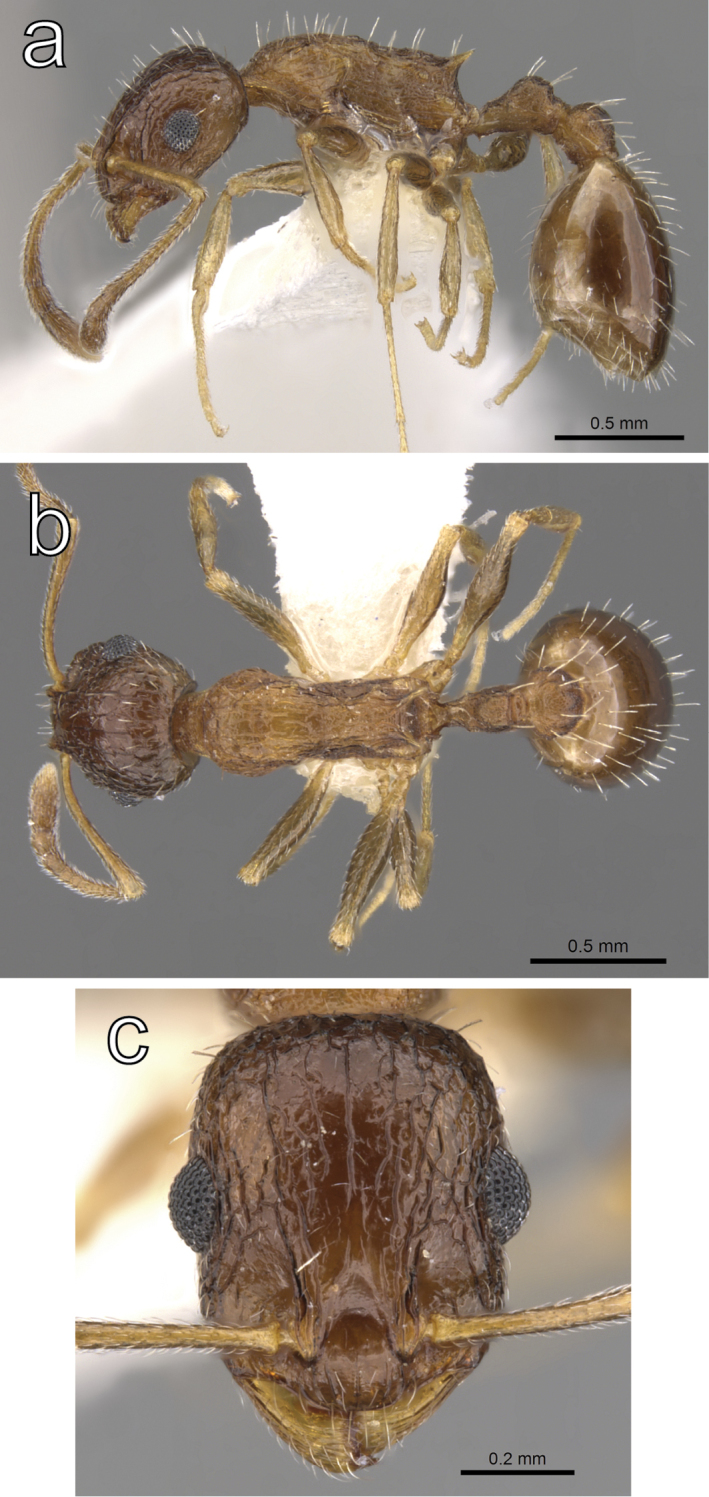
*Temnothorax cenatus* (Bolton, 1982) paratype worker (CASENT0900308) **a** body in lateral view **b** body in dorsal view **c** head in full face view. Photographer: Zach Lieberman.

##### Queen.

Unknown

##### Male.

Unknown

##### Color.

Worker: Overall light reddish brown, with head and gaster slightly darker.

##### Distribution and ecology.

So far, *Temnothorax cenatus* is only known from few localities in Central Kenya, all of which are savannah.

The holotype was collected from leaf litter, suggesting that this may be a ground nesting species.

#### Temnothorax megalops

Taxon classificationAnimaliaHymenopteraFormicidae

(Hamann & Klemm, 1967)

**Figure 7.**
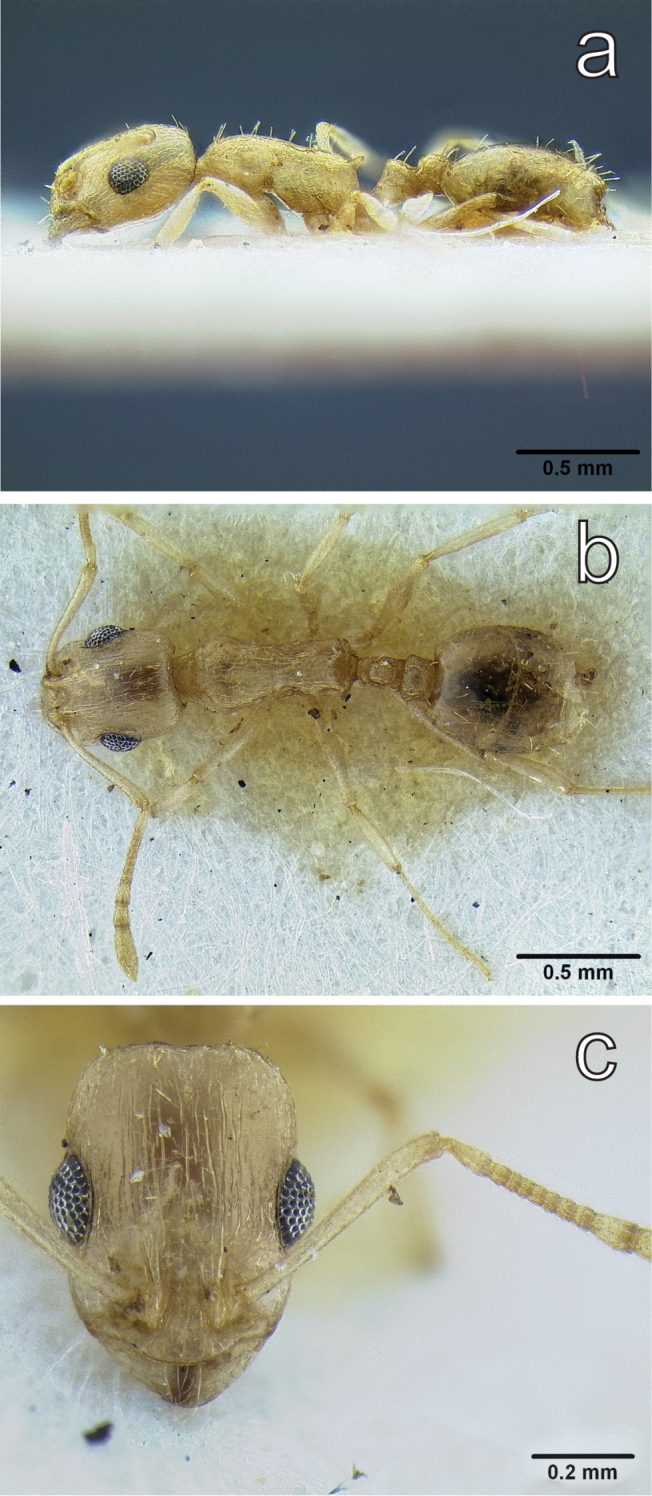
*Temnothorax megalops* (Hamman & Klemm, 1967) holotype worker (CASENT0712601) **a** body in lateral view **b** body in dorsal view **c** head in full face view.

**Figure 8.**
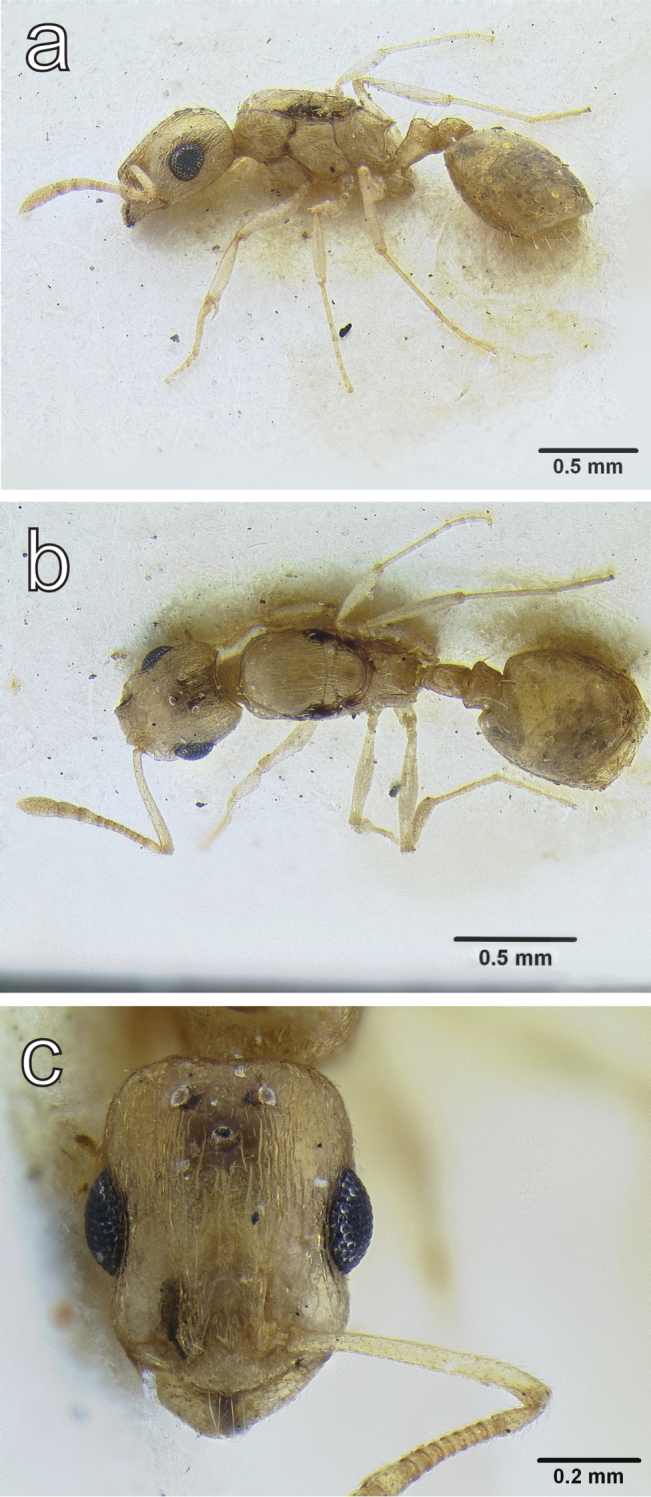
*Temnothorax megalops* (Hamman & Klemm, 1967) allotype dealate gyne (CASENT0712600) **a** body in lateral view **b** body in dorsal view **c** head in full face view.

Figs 3a and c , 7 , 8

Leptothorax megalops : Hamann and Klemm 1967: 417.
Temnothorax megalops (Hamann & Klemm): Bolton 2003: 271

##### Type material.

Holotype worker, SUDAN, Wadi Halfa, feuchter Graben, 28.I.1962 (*H. Hamann & W. Klemm*) (NHMW: CASENT0712601) [examined]. Paratype, 1 dealate queen with same data as holotype (NHMW: CASENT0712600) [examined].

##### Diagnosis.

The following character combination clearly separates *Temnothorax megalops* from the other Afrotropical *Temnothorax* species: Antennal scapes short, distinctly failing to reach the posterior margin of the head; compound eyes large; post petiole trapezoidal in dorsal view, widest at the anterior 1/5 of the segment; metanotal groove present; head subrectangular; posterior margin of head shallowly but distinctly impressed; setae on the posterior margin of the first gastric tergite separated by about their own length; pilosity of antennal scapes short and adpressed.

##### Worker measurements

**(n = 1).** EL 0.174; FRS 0.175; HL 0.636; HW 0.465; IOD 0.376; IOcD N/A; MD 0.141; PH 0.183; PL 0.208; PPH 0.178; PPL 0.127; PPW 0.192; PTW 0.139; PW 0.327; SL 0.505; SPST 0.145; WL 0.728.

**Indices:** CI 73.1; DPeI 66.8; DPpI 151; LPeI 114; LPpI 71.3; OI 37.4; PeNI 42.5; PpNI 58.7; PPI 138; PSLI 22.8; SI 79.4.

##### Worker description.

Head subrectangular, longer than wide (CI 73.1); head sides parallel, but converging toward the mandibular insertions anteriorly beyond the level of the antennal insertions in full-face view; posterior head margin with a broad, shallow median impression; posterior corners of head broadly rounded. Anterior clypeal margin convex and entire, with the median clypeal lobe projecting slightly beyond the lateral clypeal lobes. Frontal carinae poorly developed: extending posteriorly about one-quarter the length of the compound eye. Antennae 12-segmented, antennal scapes relatively short, failing to reach the posterior margin of the head (SI 109). Eyes large (OI 37.4); 11 ommatidia in the longest row.

Mesosoma relatively compact (WL 1.14 times HL); promesonotal suture absent. Metanotal groove shallowly but distinctly impressed; visible as a shallow concavity in lateral view, and as a narrowing of the dorsal surface of the mesosoma in dorsal view. Propodeal declivity steep. Propodeal spines blunt and relatively short (PSLI 22.8); propodeal lobes small and rounded.

Petiole without a differentiated peduncle. In profile, the anterior face of node forming a shallow concavity anteriorly as it joins the anterior portion of the petiole. Petiolar node in profile relatively low and cuneate (LPeI 113), junction of anterior and posterior faces forming a 90° angle; without differentiated dorsal and posterior faces. In dorsal view petiole elongate (DPeI 66.8). Postpetiole in profile with proximal half of dorsal margin evenly rounded, and distal half forming an even declivity; nearly equal in height to petiolar node and laterally compressed (LPpI 71.3). In dorsal view postpetiole trapezoidal and wider than long (DPpI 151), widest at 1/4 of the total postpetiole length from anterior margin, and 1.4 times wider than petiole (PPI 138).

Mandibular sculpture: longitudinally irregularly striate along entire length. Clypeus smooth and shiny, bearing 5 longitudinal rugae, with median ruga strongly developed and running posteriorly from the anterior clypeal margin to the level of antennal insertions before weakening. Cephalic dorsum smooth and shiny with weak, closely spaced longitudinal rugae, becoming sparse medially. In profile, gena anterior to the compound eye reticulate but otherwise similar to sculpture on dorsum. Sculpture of mesosoma in dorsal view punctate, becoming weak on pronotum, with overlying weak, longitudinal rugae which become stronger in the metanotal suture. Space between propodeal spines with a single fine, arcuate transverse carina, which divides the propodeal dorsum from the declivity. Propodeal declivity shallowly punctate and shining. In profile, mesosoma predominantly longitudinally rugose; humeri weakly reticulate, propodeum finely punctate. Petiole and postpetiole finely punctate, with a couple of weak longitudinal rugae visible on dorsum of postpeiole. Gaster smooth and shining except for small, widely spaced piligerous punctures.

Mandibles and ventral region of the head with a short, fine yellowish pubescence. Posterior margin of clypeus with two pairs of short, stout setae flanking each side of the median lobe. Frontal carinae with one stout seta each, located at the level of the anterior margin of the compound eye. Posterior margin of the head equipped with several short, blunt-tipped setae. Anterior clypeal margin with two pairs of long setae flanking the median carina. Scapes with abundant, short, subdecumbant pilosity. Propleurae with several short, sharp-tipped setae. Pronotal “neck” and pronotal humeri, and procoxae free of pubescence. Dorsal surfaces of mesosoma, waist segments and gaster with uniformly erect, short, sparse and blunt-tipped bristle-like setae; their bases spaced from each other by more than the length of the setae which arise from them. Ventral surfaces of the post-petiole and gaster free of pilosity.

##### Queen measurements

**(n = 1).** EL 0.205; FRS 0.204; HL 0.661; HW 0.506; IOD 0.407; IOcD 0.135; MD 0.147; PH 0.21; PL 0.233; PPH 0.177; PPL 0.153; PPW 0.241; PTW 0.177; PW 0.422; SL 0.536; SPST 0.188; WL 0.904.

**Indices:** CI 76.6; DPeI 76; DPpI 158; LPeI 111; LPpI 86.4; OI 40.5; PeNI 41.9; PpNI 57.1; PPI 136; PSLI 28.4; SI 81.1.

##### Queen description.

Head subrectangular, longer than wide (CI 76.6); head sides parallel, but converging toward the mandibular insertions anteriorly beyond the level of the antennal insertions in full-face view; posterior head margin with a broad, shallow median impression and posterior corners of head broadly rounded. Anterior clypeal margin convex and entire, with the median clypeal lobe projecting slightly beyond the lateral clypeal lobes. Frontal carinae poorly developed: extending posteriorly about one-half the length of the compound eye. Antennae 12-segmented,antennal scapes relatively short, failing to reach the posterior margin of the head (SI 106). Eyes large (OI 40.5); 13 ommatidia in the longest row. Three ocelli present.

Body more massive than the worker; mesosoma somewhat elongate (WL 1.37 times HL). Scutum and scutellum forming an even, flat surface in profile, broken only by the suture between the two tergites. Propodeal declivity steep. Propodeal spines blunt and slightly longer than in the worker (PSLI 28.4); propodeal lobes small and rounded.

Petiole without a differentiated peduncle. In profile, the anterior face of node forming a shallow concavity anteriorly as it joins the anterior portion of the petiole. Petiolar node in profile relatively low and cuneate (LPeI 111), junction of anterior and posterior faces forming a 90° angle; without differentiated dorsal and posterior faces. In dorsal view petiole elongate (DPeI 76). Postpetiole in profile with proximal half of dorsal margin evenly rounded, and distal half forming an even declivity; nearly equal in height to petiolar node and laterally compressed (LPpI 86.4). In dorsal view postpetiole trapezoidal and wider than long (DPpI 158); widest in the anterior 1/4, and 1.4 times wider than petiole (PPI 136).

Mandibular sculpture: longitudinally irregularly striate along entire length. Clypeus smooth and shiny, bearing 5 longitudinal rugae, with median ruga strongly developed and running posteriorly from the anterior clypeal margin to the level of antennal insertions before weakening. Cephalic dorsum with closely spaced longitudinal rugae, extending the entire length of the head, but becoming weak between the compound eyes and ocelli. In profile, gena anterior to the compound eye strongly reticulate. Scutum and scutellum with longitudinal rugae; propodeum reticulate. Space between propodeal spines with a single strong, arcuate transverse carina, which divides the propodeal dorsum from the declivity. Propodeal declivity with longitudinal rugae. In profile, mesosoma predominantly longitudinally rugose; anterior of pronotum reticulate; sculpture weakened on mesopleuron. Petiole and postpetiole finely punctate, with longitudinal rugae on dorsum of postpeiole. Gaster smooth and shining except for small, widely spaced piligerous punctures.

Mandibles and ventral region of the head with a short, fine yellowish pubescence. Posterior margin of clypeus with two pairs of short, stout setae flanking each side of the median lobe. Frontal carinae with one stout seta each, located at the level of the anterior margin of the compound eye. Dorsal surface of head equipped with several short, blunt-tipped setae. Anterior clypeal margin with two pairs of long setae flanking the median carina. Scapes with abundant, short, subdecumbant pilosity. Propleurae with several short, sharp-tipped setae. Pronotal “neck” and pronotal humeri with short, stout and sparse setae.Procoxae with short, thin and sparse setae. Dorsal surfaces of mesosoma, waist segments and gaster with uniformly erect, sparse and blunt-tipped bristle-like setae which is longer than in the worker. Ventral surfaces of the post-petiole free of pilosity; ventral surface of gaster with short, fine, yellowish pilosity.

##### Male.

Unknown

##### Color.

Worker:Yellow overall with gaster slightly infuscated at the posterior margin of the first tergite.

Queen: Same as worker.

##### Distribution and ecology.

Known only from the type material, which was collected from Wadi Halfa, Sudan.

#### Temnothorax mpala

Taxon classificationAnimaliaHymenopteraFormicidae

Prebus sp. n.

http://zoobank.org/1C409951-D8D2-4FD6-B0BD-D4577BD54284

**Figure 9.**
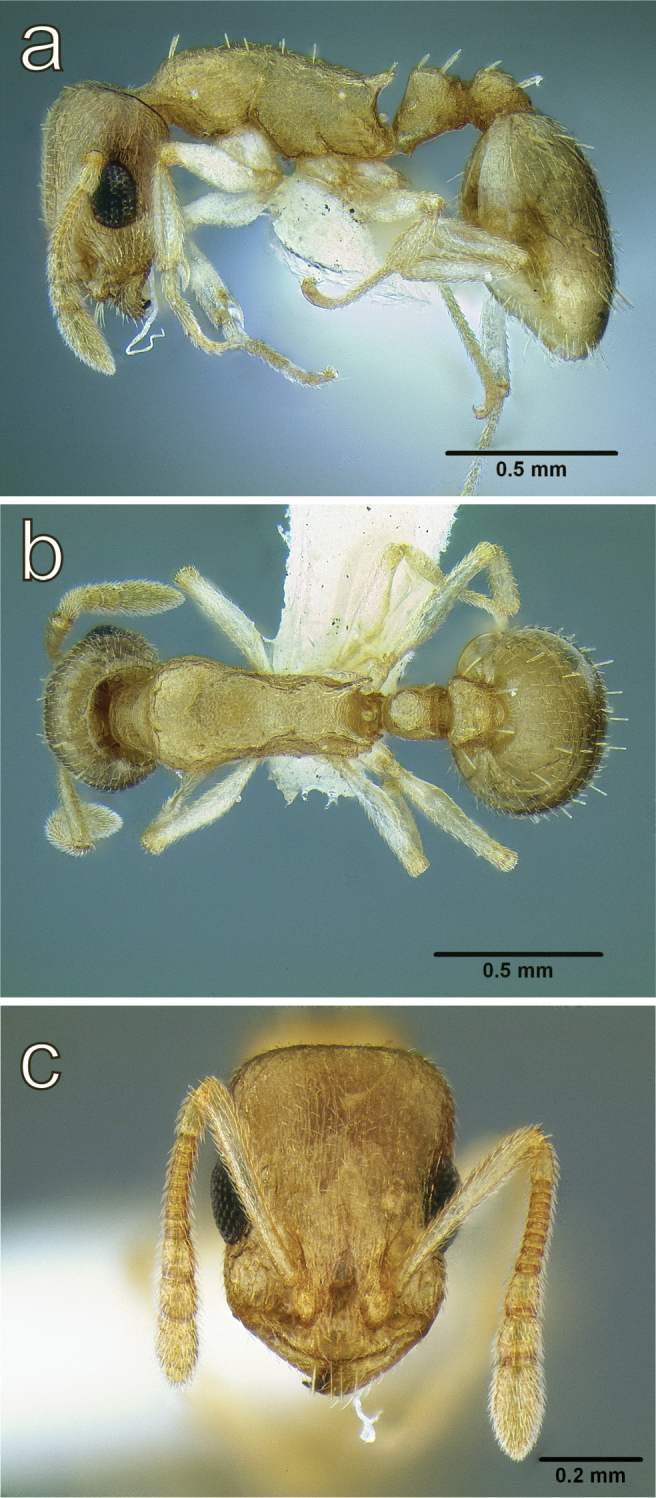
*Temnothorax mpala* sp. n., worker (LACMENT323183) **a** body in lateral view **b** body in dorsal view **c** head in full face view.

**Figure 10.**
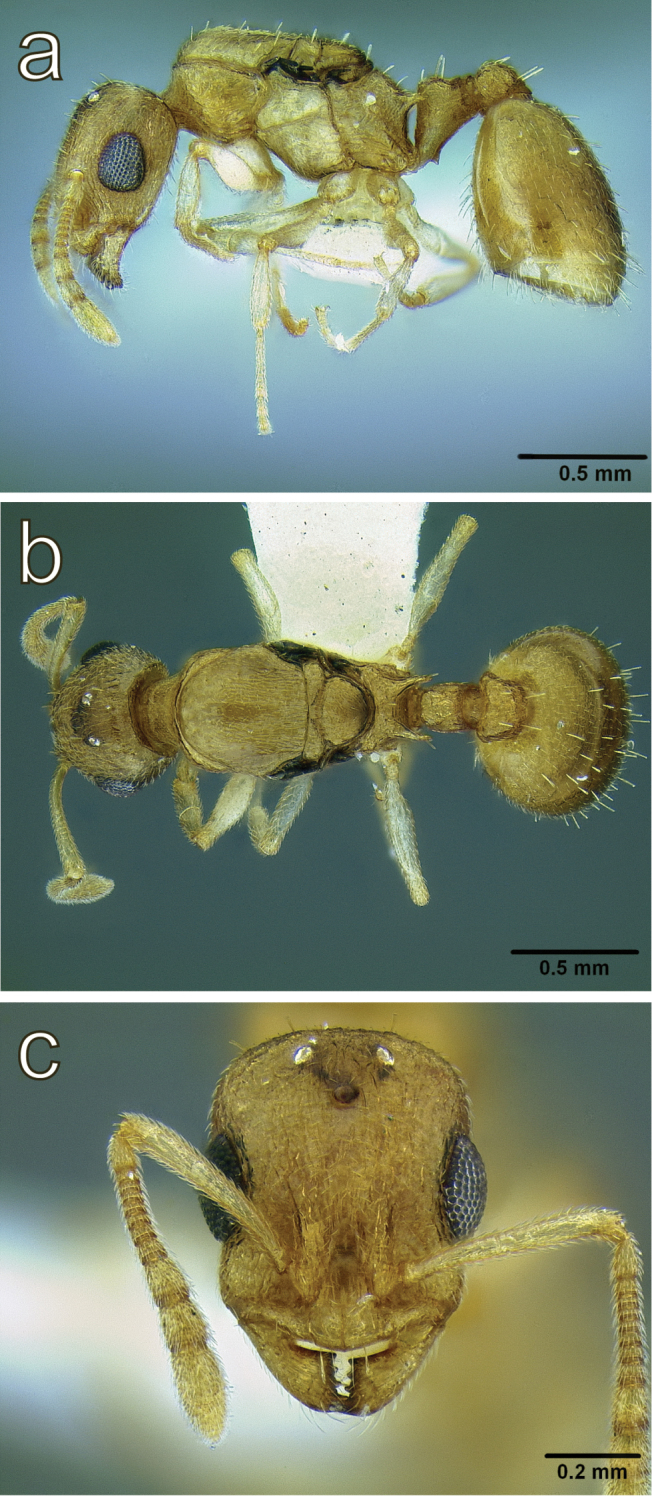
*Temnothorax mpala* sp. n., dealate gyne (LACMENT323183) **a** body in lateral view **b** body in dorsal view **c** head in full face view.

**Figure 11.**
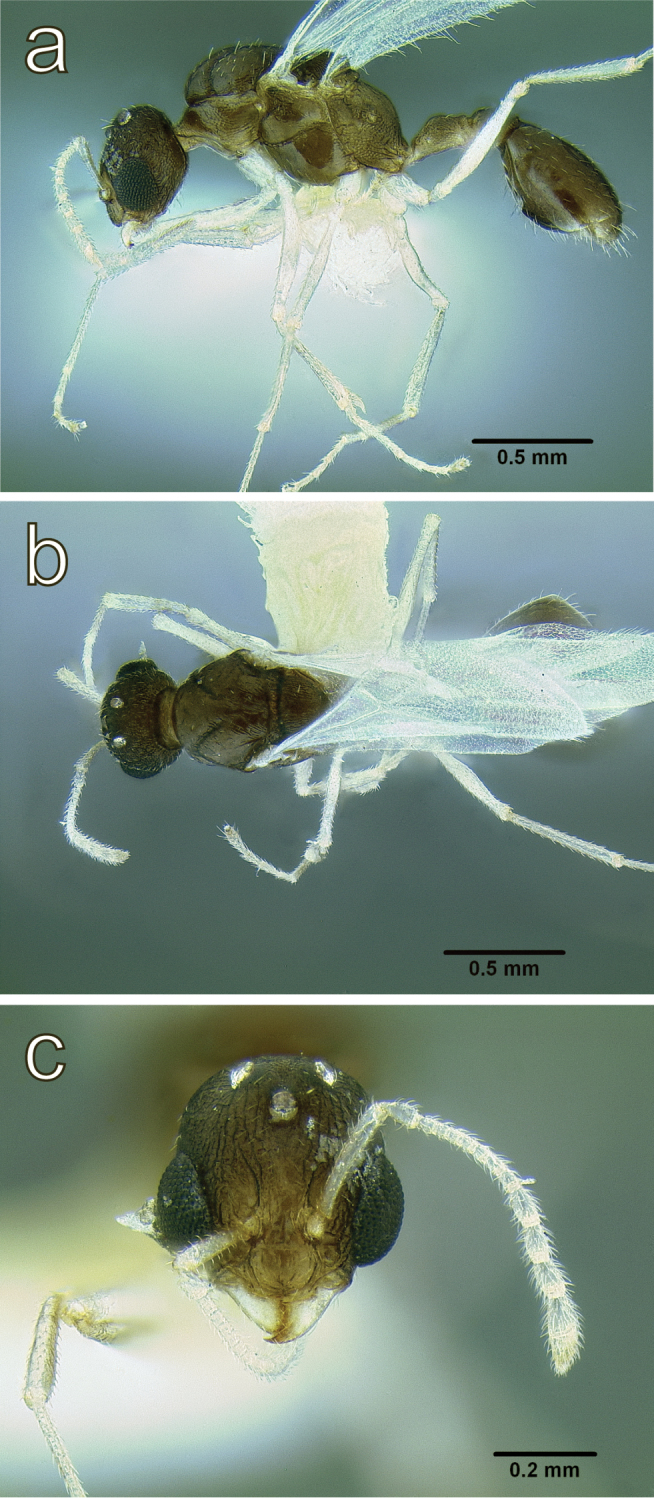
*Temnothorax mpala* sp. n., male (LACMENT323186) **a** body in lateral view **b** body in dorsal view **c** head in full face view.

Figs 1 , 2a and c , 3b and d , 9 , 10 , 11

##### Type material.

Holotype worker: 1 worker: KENYA, Laikipia District, Mpala Research Centre, 0.28°N, 36.87°E, 1700m, 18.III.2001 “Black Cotton” ex. pitfall trap in No. exclosure (*D. Misurelli*) (BMNH: LACMENT323183). Paratypes: 1 dealate queen: same data as previous (CASENT0733785). 1 worker, 1 dealate queen: same data as previous (NHMW: LACMENT323184). 1 male, 1 dealate queen: same data as previous (BMNH: LACMENT323185). 2 workers, 1 dealate queen: same data as previous (HLMD: LACMENT323186). KENYA, Laikipia District, Mpala Research Centre, 1650 m, 0.29°N, 36.90°E, Acacia woodland, ex pitfall trap near centre, collection code #01-225, 12.IV.2001 (*R.R. Snelling*) (BMNH: CASENT0280870). 1 worker, 1 dealate queen: same data as previous (LACM: CASENT0712602).

##### Diagnosis.

*Temnothorax mpala* is easily distinguishable from the other Afrotropical species by the following character combination:

Antennal scapes short, distinctly failing to reach the posterior margin of the head; compound eyes large; post petiole trapezoidal in dorsal view, widest at the anterior 1/5 of the segment; metanotal groove absent; head subrectangular; posterior margin of head flat; setae on the posterior margin of the first gastric tergite separated by about their own length; pilosity on antennal scape short and adpressed.

##### Worker measurements

**(n = 6).** EL 0.168–0.193 (0.185); FRS 0.175–0.19 (0.182); HL 0.598–0.67 (0.631); HW 0.445–0.488 (0.465); IOD 0.343–0.372 (0.355); IOcD N/A; MD 0.134–0.157 (0.142); PH 0.195–0.22 (0.205); PL 0.2–0.233 (0.218); PPH 0.172–0.185 (0.179); PPL 0.125–0.154 (0.142); PPW 0.216–0.239 (0.227); PTW 0.156–0.174 (0.162); PW 0.34–0.373 (0.354); SL 0.43–0.467 (0.443); SPST 0.161–0.183 (0.166); WL 0.705–0.772 (0.733).

**Indices:** CI 72.8–75.9 (73.6); DPeI 71.1–83 (74.5); DPpI 150.7–178.4 (160.7); LPeI 95–112 (107); LPpI 71.4–83.7 (79.2); OI 37–41.6 (39.8); PeNI 45–46.8 (45.9); PpNI 62.2–65.8 (64.1); PPI 134.9–145.1 (139.7); PSLI 25.4–27.3 (26.3); SI 68.3–72.6 (70.2).

##### Worker description.

Head subrectangular, longer than wide (CI 72.8–75.9); head sides parallel, but converging toward the mandibular insertions anteriorly beyond the level of the antennal insertions in full-face view; posterior head margin flat and posterior corners of head broadly rounded. Anterior clypeal margin convex and entire, with the median clypeal lobe projecting slightly beyond the lateral clypeal lobes. Frontal carinae poorly developed: extending posteriorly about one-quarter the length of the compound eye. Antennae 12-segmented; antennal scapes short, failing to reach the posterior margin of the head (SI 91.3–98.9). Eyes large (OI 37.0–41.6). with 11 ommatidia in longest row.

Mesosoma compact (WL 1.17 times HL); promesonotal suture absent. Metanotal groove not impressed, but marked by a faint narrowing of the dorsal surface of the mesosoma in dorsal view. Propodeal spines acute and relatively short (PSLI 25.4–27.3); propodeal lobes small and rounded.

Petiole without a differentiated peduncle. In profile, the anterior face of node forming a very shallow concavity anteriorly as it joins the anterior portion of the petiole. Petiolar node in profile low (LPeI 95.0–112), with dorsal and posterior faces joined by a rounded 120° angle. Subpetiolar process is in the form of a small tooth in the anterior 1/4 of the petiole. In dorsal view petiole somewhat elongate (DPeI 71.1–83.0). Postpetiole in profile with proximal half of dorsal margin evenly rounded, and distal half forming an even declivity; slightly shorter than petiolar node and laterally compressed (LPpI 71.4–83.7). In dorsal view postpetiole trapezoidal and wider than long (DPpI 151–153), widest in the anterior 1/4, and 1.4–1.5 times wider than petiole (PPI 135–145).

Mandibular sculpture: longitudinally striate along entire length. Clypeus smooth and shiny, bearing 9 longitudinal rugae, with median ruga strongly developed and running posteriorly from the anterior clypeal margin to the level of antennal insertions before weakening. Cephalic dorsum reticulate, with transverse rugae becoming weaker posterior to the level of the compound eyes. In profile, head irregularly reticulate-punctate. Sculpture of mesosoma in dorsal view punctate, with overlying weak reticulation on pronotum. Space between propodeal spines with a single extremely fine, arcuate transverse carina, which divides the propodeal dorsum from the declivity. Propodeal declivity uniformly punctate, bordered laterally by carinae which run from the ventral margins of the propodeal spines to the propodeal lobes. In profile, mesosoma predominantly irregularly punctate; humeri weakly reticulate. Petiole and postpetiole finely punctate. Gaster smooth and shining except for small, widely spaced piligerous punctures.

Head, including mandibles, with a short, sparse, adpressed fine yellowish pubescence. Mandibles with longer setae along the distal margins. Posterior margin of clypeus with one pair of short, stout setae directly below the antennal insertions. Frontal carinae with three pairs of stout setae, located at the level of the antennal insertions, anterior margin of the compound eye, and midway up the compound eye. Region of the head posterior to the level of the compound eyes equipped with three pairs of short, blunt-tipped setae: one located medially, posterior to the level of the compound eyes and two pairs on the posterior margin of the head. Anterior clypeal margin with two pairs of long setae flanking the median carina. Scapes with abundant, short subdecumbant pilosity. Pronotal “neck” with pubescence similar to that which is found on the head. Propleurae with several short, sharp-tipped setae. Procoxae with short, adpressed and abundant pilosity. Pronotal humeri free of pubescence. Dorsal surfaces of mesosoma and waist segments with uniformly erect, short, sparse and spatulate setae; their bases spaced from each other by more than the length of the setae which arise from them. All surfaces of gaster covered in sparse, evenly spaced short pilosity. Dorsal surface of first gastral tergite largely free of setae longer than the ground pilosity, save for several irregularly spaced stout setae. Anterior margins of all gastral tergites with evenly spaced short, stout setae. Ventral surfaces of the post-petiole with several short, fine setae. Ventral surface of gaster with abundant long fine pilosity.

##### Queen measurements

**(n = 4).** EL 0.241–0.248 (0.245); FRS 0.212–0.218 (0.214); HL 0.68–0.71 (0.699); HW 0.534–0.551 (0.543); IOD 0.411–0.427 (0.421); IOcD 0.139–0.148 (0.142); MD 0.131–0.143 (0.139); PH 0.232–0.259 (0.246); PL 0.242–0.29 (0.265); PPH 0.224–0.232 (0.228); PPL 0.162–0.177 (0.169); PPW 0.277–0.295 (0.283); PTW 0.192–0.207 (0.201); PW 0.484–0.501 (0.491); SL 0.459–0.489 (0.481); SPST 0.214–0.221 (0.217); WL 1.029–1.07 (1.054).

**Indices:** CI 76.8–78.5 (77.7); DPeI 71.4–83.1 (76.2); DPpI 163.9–172.8 (167.7); LPeI 98–116 (108); LPpI 71.1–76.3 (74); OI 45–45.4 (45.1); PeNI 39.7–41.8 (41); PpNI 57.1–58.9 (57.7); PPI 136.6–144.3 (140.8); PSLI 30.5–31.5 (31.1); SI 67.5–69.4 (68.8).

##### Queen description.

Head subrectangular, longer than wide (CI 76.8–78.5); head sides parallel, but converging toward the mandibular insertions anteriorly beyond the level of the antennal insertions in full-face view; posterior head margin flat; posterior corners of head more broadly rounded than in the worker. Anterior clypeal margin convex and entire, with the median clypeal lobe projecting slightly beyond the lateral clypeal lobes. Frontal carinae developed: extending posteriorly to about one-half the length of the compound eye. Antennae 12-segmented;antennal scapes short, failing to reach the posterior margin of the head (SI 86.0–89.9). Eyes large (OI 45.0–45.5). with 14–16 ommatidia in longest row. Three ocelli present.

Mesosoma more massive and elongate than in the worker (WL 1.51 times HL).Scutum and scutellum forming an even, flat surface in profile, broken only by the suture between the two tergites. Propodeal declivity steep. Propodeal spines acute and slightly longer than in the worker (PSLI 30.5–31.5); propodeal lobes small and rounded.

Petiole without a differentiated peduncle. In profile, the anterior face of node not forming a shallow concavity anteriorly as it joins the anterior portion of the petiole. Petiolar node in profile relatively low and truncate (LPeI 98.0–116), junction of anterior and dorsal faces forming a rounded 120° angle; dorsal and posterior faces differentiated by a rounded 135° angle. Subpetiolar process is in the form of a small tooth at the anterior 1/4 of the petiole. In dorsal view petiole elongate (DPeI 71.4–83.1). Postpetiole in profile with proximal half of dorsal margin evenly rounded, and distal half forming an even declivity; slightly shorter than petiolar node and laterally compressed (LPpI 71.1–76.3). In dorsal view postpetiole trapezoidal and wider than long (DPpI 164–173); widest in the anterior 1/4, and 1.4 times wider than petiole (PPI137–144).

Mandibular sculpture: longitudinally irregularly striate along entire length. Clypeus smooth and shiny, bearing 5 longitudinal rugae and a few irregular rugae on posteriorly; median ruga strongly developed and running posteriorly from the anterior clypeal margin to the level of antennal insertions before weakening. Cephalic dorsum with closely spaced longitudinal rugae, extending the entire length of the head. In profile, gena anterior to the compound eye strongly reticulate. Scutum and scutellum with light longitudinal rugae and shallow foveae, becoming smooth and polished on the anterior of the scutum in some specimens; propodeum reticulate. Space between propodeal spines with a single arcuate transverse carina, which divides the propodeal dorsum from the declivity. Propodeal declivity weakly reticulate. In profile, mesosoma predominantly longitudinally rugose; anterior of pronotum reticulate; sculpture weakening slightly on mesopleuron. Petiole and postpetiole finely punctate; dorsum of postpetiole reticulate. Gaster smooth and shining except for small, widely spaced piligerous punctures.

Head, including mandibles, with a short, sparse, adpressed fine yellowish pubescence. Mandibles with longer setae along the distal margins. Posterior margin of clypeus with one pair of short, stout setae directly below the antennal insertions. Frontal carinae with three pairs of stout setae, located at the level of the antennal insertions, anterior margin of the compound eye, and midway up the compound eye. Posterior of head equipped with three pairs of short, blunt-tipped setae; one flanking the anterior ocellus, and two pairs on posterior margin of the head. Anterior clypeal margin with two pairs of long setae flanking the median carina. Scapes with abundant, short, subdecumbant pilosity. Pronotal “neck” with pubescence similar to that which is found on the head. Propleurae with several short, fine setae.Coxae with short, thin and sparse setae. Dorsal surfaces of mesosoma, waist segments and gaster with uniformly erect, sparse and blunt-tipped bristle-like setae. Dorsum of mesosoma with fine, short, sparse yellowish setae. Ventral surface of the petiole free of pilosity, but post-petiole has a pair of hairs latero-ventrally. All surfaces of gaster with short, fine, yellowish pilosity which becomes longer ventrally.

##### Male measurements

**(n = 1).** EL 0.222; FRS 0.123; HL 0.498; HW 0.419; IOD 0.271; IOcD 0.131; MD 0.044; PH 0.151; PL 0.22; PPH 0.151; PPL 0.142; PPW 0.196; PTW 0.14; PW 0.487; SL 0.262; SPST N/A; WL 0.957.

**Indices:** CI 84.1; DPeI 63.6; DPpI 138; LPeI 146; LPpI 94; OI 53; PeNI 28.7; PpNI 40.2; PPI 140; PSLI N/A; SI 52.6.

##### Male description.

Head globular, small and somewhat longer than wide (CI 84.1); head sides rounding broadly into the posterior of the head. Anterior clypeal margin convex and entire, with the median clypeal lobe projecting strongly beyond the lateral clypeal lobes. Frontal carinae developed: extending posteriorly to the posterior margin of the compound eye. Antennae 13-segmented, with a four-segmented club;antennal scapes very short, failing to reach the posterior margin of the head (SI 62.5). Eyesvery large (OI 53) and close to the mandibular insertions (MD 0.044). 20 ommatidia in longest row. Three ocelli present.

Mesosoma more massive and elongate than in the worker (WL 1.92 times HL).Scutum and scutellum forming an even, flat surface in profile, broken only by the suture between the two tergites. Dorsal surface of propodeum rounding evenly into the propodeal declivity. Propodeal spines absent.

Petiole without a differentiated peduncle. In profile, petiolar node indistinct, forming a low mass (LPeI 146). Subpetiolar process reduced to a small bump in the anterior 1/4 of the petiole in profile. In dorsal view petiole elongate (DPeI 63.6). Postpetiole in profile with proximal half of dorsal margin evenly rounded, and distal half forming an even declivity; slightly shorter than petiolar node (LPpI 94). In dorsal view postpetiole weakly trapezoidal and slightly wider than long (DPpI 138);slightly wider in the anterior half, and 1.4 times wider than petiole (PPI140).

Mandibular sculpture: smooth and shiny along their entire length. Clypeus smooth and shiny, bearing several irregular rugae. Cephalic dorsum with closely spaced longitudinal rugae with slightly weaker reticulations and punctuations between them. Scutum and scutellum with light longitudinal rugae, becoming smooth and polished on the anterior half of the scutum; propodeum weakly reticulate over a punctate ground sculpture. Propodeal declivity weakly punctate. In profile, mesosoma predominantly smooth and shiny; anterior of pronotum, dorsal margin of the anepisternum and propodeum weakly punctate. Petiole and postpetiole finely punctate; dorsum of postpetiole smooth and weakly punctate. Gaster smooth and shining except for small, widely spaced piligerous punctures.

Head, including mandibles, with a short, sparse, adpressed fine yellowish pubescence. Mandibles with longer setae along the distal margins. Posterior margin of clypeus with one pair of short, stout setae directly below the antennal insertions. One pair of short, blunt-tipped setae flank the anterior ocellus. Scapes with abundant, short, subdecumbant pilosity. Pronotal “neck” with pubescence similar to that which is found on the head. Propleurae with several short, fine setae.Coxae with short, thin and sparse setae. Dorsal surfaces of mesosoma and waist segments with uniformly erect, sparse and blunt-tipped bristle-like setae. Dorsum of mesosoma with fine, short, and very sparse setae. Ventral surface of the petiole free of pilosity, but post-petiole has a pair of hairs latero-ventrally. All surfaces of gaster with fine, yellowish, sparse pilosity which becomes longer ventrally.

##### Color.

Worker: Overall light brownish-yellow, with slightly darker head and gaster and lighter legs (including coxae).

Queen: Same as worker, but with dorsum of mesosoma same color as head and mesosoma.

Male: Overall dark reddish brown, with ventral surfaces of waist segements slightly lighter. Extemities, including antennae, mouthparts, legs and coxae cream-colored.

##### Distribution and ecology.

*Temnothorax mpala* is only known from the leaf litter of acacia woodland at the type locality, Mpala Research Centre. Curiously, workers, dealate gynes as well as one male were recovered from pitfall traps, suggesting that these collection events captured a nest migration.

##### Taxonomic notes.

The word “mpala” comes from a Bantu name for a type of antelope that was kept by chiefs of the Bunganda kingom. *Temnothorax mpala* is not expected to be particularly swift in its movements; the only known specimens were collected at the above mentioned Mpala Research Centre in Laikipia, Kenya.

#### Temnothorax rufus

Taxon classificationAnimaliaHymenopteraFormicidae

Prebus sp. n.

http://zoobank.org/F2293A69-0C37-4A51-9095-BAAEFBD1B0D3

**Figure 12.**
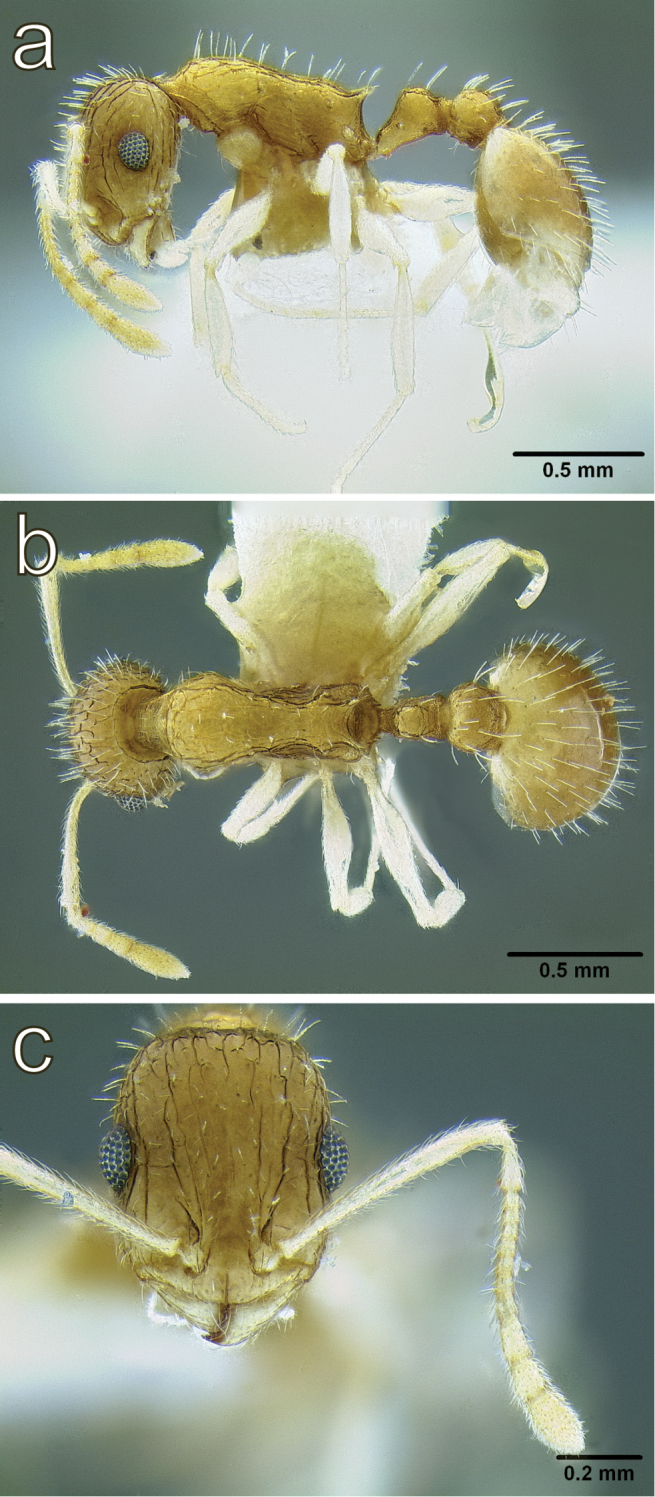
*Temnothorax rufus* sp. n., worker (CASENT0712675) **a** body in lateral view **b** body in dorsal view **c** head in full face view.

Figs 2b and d , 4c , 12

##### Type material.

Holotype worker, KENYA, Kora National Park, “Salvadora pitfall”, collection code no. 18, 1983 N.M. Collins & M. Ritchie (BMNH: CASENT0712675). Paratype, 1 worker with same data as holotype (HLMD: CASENT0733784).

##### Diagnosis.

*Temnothorax rufus* is easily distinguishable from the other Afrotropical species by the following character combination:

Antennal scapes surpassing the posterior margin of the head by the length of the first two funicular segments; postpetiole widest at the anterior 1/3 of the segment;posterior margin of head rounded; promesonotal suture shallowly impressed; compound eyes moderate in size; propodeal spines moderately long.

##### Worker measurements

**(n = 2).** EL 0.162–0.164 (0.163); FRS 0.191–0.196 (0.194); HL 0.654–0.664 (0.659); HW 0.517–0.527 (0.522); IOD 0.446–0.455 (0.451); IOcD N/A; MD 0.167–0.18 (0.174); PH 0.19–0.213 (0.202); PL 0.286–0.299 (0.293); PPH 0.21–0.222 (0.216); PPL 0.204–0.211 (0.208); PPW 0.257–0.278 (0.268); PTW 0.156–0.176 (0.166); PW 0.375–0.386 (0.381); SL 0.626–0.633 (0.63); SPST 0.169–0.177 (0.173); WL 0.863–0.868 (0.866).

**Indices:** CI 79.1–79.4 (79.2); DPeI 54.5–58.9 (56.7); DPpI 122–136 (129); LPeI 140–151 (145); LPpI 91.9–100.5 (96.2); OI 31.1–31.3 (31.2); PeNI 41.6–45.6 (43.6); PpNI 68.5–72 (70.3); PPI 158–165 (161); PSLI 25.8–26.7 (26.2); SI 94–96.8 (96).

##### Worker description.

Head longer than wide (CI 79.1–79.4); head sides parallel, but converging toward the mandibular insertions anteriorly beyond the level of the antennal insertions in full-face view; posterior head margin broadly convex and posterior corners of head broadly rounded. Anterior clypeal margin convex, with the median clypeal lobe projecting slightly beyond the lateral clypeal lobes. Frontal carinae developed: extending posteriorly to about midlength of the compound eye, after which they become indistinguishable from the ground rugulae of the head. Antennae 12-segmented,antennal scapes long, surpassing the posterior margin of the head by about the length of the first two funicular segments (SI 119–122). Eyes moderate in size (OI 31.1–31.3), 11 ommatidia in longest row.

Mesosoma relatively slender (WL 1.31–1.32 times HL); promesonotal suture not impressed. Metanotal groove shallowly impressed; visible as a broad, shallow concavity in lateral view. Propodeal spines acute and moderately long (PSLI 25.8–26.7); propodeal lobes small and rounded.

Petiole without a differentiated peduncle. In profile, petiole with a low carina running transversely from the petiolar spiracle to the posterior margin; the anterior face of node forming a shallow concavity anteriorly as it joins the anterior portion of the petiole. Petiolar node in profile relatively low, with anterior and posterior faces broadly rounded (LPeI 140–151). In dorsal view petiole elongate (DPeI 54.5–58.9). Postpetiole in profile globular, nearly equal in height to petiolar node and relatively elongate (LPpI 91.9–100.5); in dorsal view transversely elongate-oval, widest at 1/3 of the total postpetiole length from anterior margin (DPpI 122–136) and 1.6 times wider than petiole (PPI 158–165).

Mandibular sculpture: distinctly longitudinally striate along entire length. Clypeus smooth and shiny, bearing 9 longitudinal rugae, with median ruga strongly developed and running posteriorly from the anterior clypeal margin to the level of antennal insertions before weakening. Cephalic dorsum predominantly longitudinally rugose, with transverse rugae incompletely connecting longitudinal rugae. In profile, sides of head coarsely rugo-reticulate; coarse punctures visible between rugae, particularly postero-ventrally to the compound eye. Sculpture of mesosoma in dorsal view with predominately longitudinal rugae on pronotum and mesonotum, becoming increasingly reticulate on the propodeum. Space between propodeal spines with several fine transverse rugae, propodeal declivity finely punctate. In profile, mesosoma rugo-reticulate; longitudinal rugae stronger on pronotum, becoming increasingly reticulate on mesopleuron, and giving way to coarse punctation on metapleuron. Petiole and postpetiole finely punctate, with weak overlying rugosity. Gaster smooth and shining except for small, widely spaced piligerous punctures.

Head, including mandibles and ventral regions nearly uniformly covered in a fine, whitish pubescence. Dorsal surface of the head, including clypeus, frons, posterior margin of the head and occipital corners equipped with long, blunt-tipped setae. Anterior clypeal margin with two pairs of long setae flanking the median carina. Antennal scape pilosity abundant, sharp-tipped and subdecumbent. Pronotal “neck”, pronotal humeri, propleurae and upper half of procoxae with short, fine whitish pubescence. Dorsal surfaces of mesosoma, waist segments and gaster with uniformly erect, long, abundant, blunt-tipped whitish setae, their bases spaced from each other by the length of the setae or less. Bases of the setae on the posterior margin of first gastral tergite separated by less than the length of the setae. Ventral surfaces of the post-petiole and gaster with sparse pilosity like that of the propleuron.

##### Queen.

Unknown

##### Male.

Unknown

##### Color.

Worker: Overall yellowish-orange with extremities, including antennae, legs, coxae, mouthparts and gaster (exluding first tergite) yellowish-white.

##### Distribution and ecology.

*Temnothorax rufus* is known only from the type locality, Kora National Park, which includes acacia bushland and riverine forests. The type specimens were collected via pitfall traps; presumably this is a ground-nesting species.

##### Taxonomic notes.

The only known specimens of *Temnothorax rufus* exhibit coloration unique among the species of the Afrotropical region, having light orange head, mesosoma and gaster and pale extremities.

#### Temnothorax solidinodus

Taxon classificationAnimaliaHymenopteraFormicidae

Prebus sp. n.

http://zoobank.org/6BC6BA04-F0E3-4714-957F-B62A2D7D60A4

Fig. 13

##### Type material.

Holotype worker: KENYA, Kakamega Forest, Colobus. 0.27°N, 34.88°E, 1600 m. Rainforest, canopy fogging *Teclea nobilis* “101”. x.2002. leg. W. Freund. (HLMD: CASENT0906153).

##### Diagnosis.

*Temnothorax solidinodus* is easily distinguishable from the other Afrotropical species by the following character combination:

Antennal scapes short, distinctly failing to reach the posterior margin of the head; compound eyes moderate in size (OI < 30); post petiole globular in dorsal view, widest at the midpoint of the segment; metanotal groove shallowly impressed; head subrectangular (CI > 85); posterior margin of head flat.

##### Worker measurements

**(n = 1).** EL 0.185; FRS 0.257; HL 0.728; HW 0.664; IOD 0.575; IOcD N/A; MD 0.153; PH 0.295; PL 0.298; PPH 0.248; PPL 0.198; PPW 0.265; PTW 0.215; PW 0.45; SL 0.507; SPST 0.232; WL 0.921.

**Indices:** CI 91.2; DPeI 72.1; DPpI 134; LPeI 101; LPpI 79.8; OI 27.9; PeNI 47.8; PpNI 58.9; PPI 123; PSLI 31.9; SI 69.6.

##### Worker description.

Head sub rectangular (CI 91.2); head sides converging toward the mandibular insertions anterior to the compound eyes in full-face view; posterior margin of head flat, occipital corners rounded. Anterior clypeal margin slightly concave medially, with the median clypeal lobe rounding evenly into the lateral clypeal lobes. Frontal carinae developed: extending posteriorly to about midlength of the compound eye, after which they become indistinguishable from the ground rugulae of the head. Antennae 12-segmented, antennal scapes short, falling short of the posterior margin of the head by about the length of the first funicular segment (SI 69.6). Eyes moderate in size (OI 27.9), 13 ommatidia in longest row.

Mesosoma relatively slender (WL 1.27 times HL); promesonotal suture very shallowly impressed and marked a distinct break in sculpture. Metanotal groove shallowly impressed; visible as a broad, shallow concavity in lateral view and lending a slightly peaked appearance to the propodeum. Propodeal spines long, straight and acute (PSLI 31.9); propodeal lobes small and rounded.

Petiole without a differentiated peduncle. In profile, the anterior face of node sloping evenly into the anterior portion of the petiole. Petiolar node in profile low, with anterior and posterior faces broadly rounded (LPeI 79.8). In dorsal view petiole elongate (DPeI 72.1). Postpetiole in profile globular, nearly equal in height to petiolar node, laterally compressed (LPpI 79.8); in dorsal view transversely elongate-rectangular, widest at the midpoint (DPpI 134) and 1.2 times wider than petiole (PPI 123).

Mandibles distinctly longitudinally striate along their entire length. Clypeus smooth and shiny, bearing 13 longitudinal rugae which become weaker medially. Cephalic dorsum predominantly longitudinally rugose, overlying punctate sculpture that weakens medially. In profile, sides of head coarsely reticulate between the compound eye and mandible. Sculpture of mesosoma in dorsal view with predominately longitudinal rugae on pronotum; mesonotum punctate on the anterior half, weakening the posterior half; propodeum predominantly longitudinally rugose. Space between propodeal spines strongly punctate, propodeal declivity with several weak transverse rugae. In profile, mesosoma, petiole and post petiole strongly rugo-reticulate over a punctate ground sculpture. Gaster smooth and shining.

Dorsal surface of the head, including clypeus, frons, posterior margin of the head and occipital corners equipped with short (less than the length of the pedicel), yellowish, blunt-tipped setae. Anterior clypeal margin with two pairs of long setae flanking the median carina. Antennal scape pilosity abundant, sharp-tipped and subdecumbent. Pronotal “neck” and procoxae with short, fine yellowish pubescence. Dorsal surfaces of mesosoma, waist segments and gaster with uniformly erect, short, abundant, blunt-tipped yellowish setae, their bases spaced from each other by the length of the setae or more. Bases of the setae on the posterior margin of first gastral tergite separated by more than the length of the setae.

Dorsal surface of head, excluding the antennae, clypeus and mandibles, dark brown. Antennae, clypeus, mandibles, mesosoma and waist segments uniformly light brown. Gaster transitions from light brown to dark brown along the anteroposterior axis.

**Figure 13.**
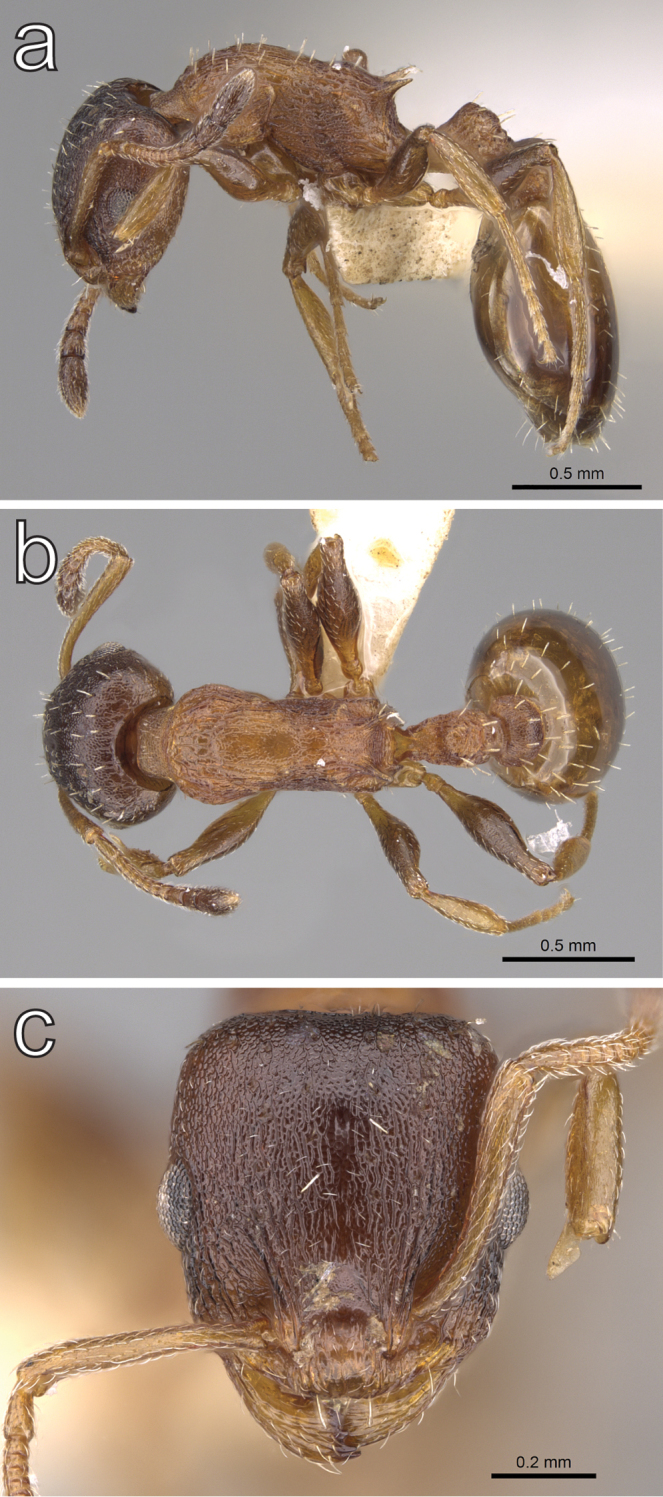
*Temnothorax solidinodus* sp. n., worker (CASENT0906153) **a** body in lateral view **b** body in dorsal view **c** head in full face view. Photographer: Michele Esposito.

##### Queen.

Unknown

##### Male.

Unknown

##### Distribution and ecology.

One worker of *Temnothorax solidinodus* was collected via canopy fogging of *Teclea nobilis* in Kakamega Forest. Presumably this is an arboreal species.

**Figure 14.**
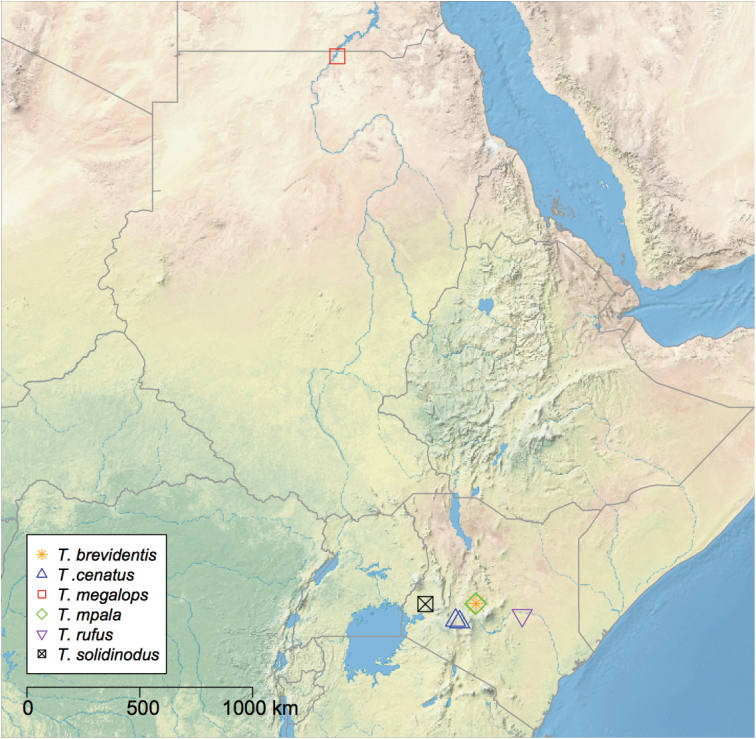
Distribution of the Afrotripical species of *Temnothorax*. Area depicted ranges from southern Egypt to northern Tanzania.

**Figure 15.**
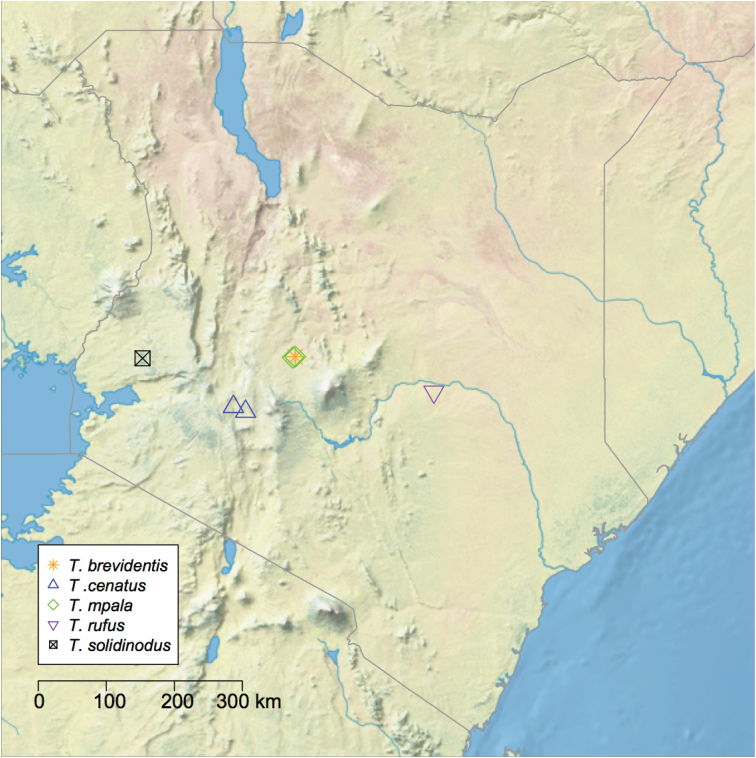
Detail of the distribution of *Temnothorax* species in sub-Saharan Africa. Area depicted includes Kenya and bordering countries

##### Color.

Worker: Overall chestnut-brown, with head and gaster posterior to the midlength of the first tergite darker.

##### Taxonomic notes.

*Temnothorax solidinodus*, in relation to *Temnothorax angustulus* and other species within the *angustulus* group bears a very large petiolar node.

## Supplementary Material

XML Treatment for Temnothorax brevidentis

XML Treatment for Temnothorax cenatus

XML Treatment for Temnothorax megalops

XML Treatment for Temnothorax mpala

XML Treatment for Temnothorax rufus

XML Treatment for Temnothorax solidinodus
